# Numerical Investigation of Pressure Profile in Hydrodynamic Lubrication Thrust Bearing

**DOI:** 10.1155/2014/157615

**Published:** 2014-10-29

**Authors:** Farooq Ahmad Najar, G. A. Harmain

**Affiliations:** Department of Mechanical Engineering, National Institute of Technology, Srinagar 190006, India

## Abstract

Reynolds equation is solved using finite difference method (FDM) on the surface of the tilting pad to find the pressure distribution in the lubricant oil film. Different pressure profiles with grid independence are described. The present work evaluates pressure at various locations after performing a thorough grid refinement. In recent similar works, this aspect has not been addressed. However, present study shows that it can have significant effect on the pressure profile. Results of a sector shaped pad are presented and it is shown that the maximum average value of pressure is 12% (approximately) greater than the previous results. Grid independence occurs after 24 × 24 grids. A parameter “*ψ*” has been proposed to provide convenient indicator of obtaining grid independent results. *ψ* = |(*P*_refinedgrid_ − *P*_Refrence-grid_)/*P*_refinedgrid_|, *ψ* ≤ *ε*, where “*ε*” can be fixed to a convenient value and a constant minimum film thickness value of 75 *μ*m is used in present study. This important parameter is highlighted in the present work; the location of the peak pressure zone in terms of (*r*, *θ*) coordinates is getting shifted by changing the grid size which will help the designer and experimentalist to conveniently determine the position of pressure measurement probe.

## 1. Introduction

In this fluid lubrication, two mating surfaces are separated by a layer of lubricant. In order to have a load carrying (positive) pressure, the film needs to be convergent in space. Consequent determination of pressure profile numerically is an important issue and values so obtained need to be checked stringently as a function of grid size. It is the pressure distribution that balances the weight of the heavy shaft and the turbine, found in hydropower generating plants with a turbine assembly. The Reynolds equation, which is derived from the Navier-Stokes (NS) equations using thin-film assumptions, is extensively used in tribological applications. The Reynolds equation in polar form can be easily found in multiple text books, if any of these sources, however, derive the polar Reynolds equation directly from the cylindrical NS equation [1–3]. The Reynolds equation is a simplified from the NS equation when analyzing a thin lubricant flow, Reynolds equation is commonly used for its practical application while NS full equations are used to find validity limits of Reynolds equation. Both methods give similar results when working with narrow gaps; however when the minimum distance of the channel throat is increased the pressure values obtained become quite different [4]. It is assumed that the fluid flow between pad and collar is never turbulent and the model applied is only valid for laminar fluids. It is a common assumption although in some cases turbulent flow exists under certain points of operation [5]. The transition from laminar to turbulent flow occurs at the leading edge first where the fluid flow is thicker. Although it is known that the results would be more accurate using the full NS equations, the complexity of the calculations is increased heavily; so the Reynolds equation has been used for the thin lubricant film calculations in several research papers. The fluid density and the viscosity are some of the significant parameters. To obtain desired pressure it is often easier to switch the lubricant type instead of modifying other parameters as the gap height or the relative motion between surfaces, which in this case is identical to the collar velocity.

The effects of pad curvatures on thrust bearing performances have been reported in [6]. It has been shown by [7, 8] that the film shapes have considerable influence on the bearing performances. It has been investigated by [9] that the effects of continuous circumferential surface profiles can be signified on the performance characteristics of a sector-type thrust bearing. As per study of [10, 11] that as compared with conventional taper fluid film shape, new surface profile (cycloidal, catenoidal, exponential, polynomial) are found to offer a significant increase in the load-carrying capacity as well as a considerable reduction in the coefficient of friction. In recent works as well, researchers have not presented the effect of grid size on the solution of Reynolds equation, for example, the work presented by [12] is cited as one of such cases, in which it is directly presented a 9 × 9 grid and has not shown effect of coarsening or refining mesh on the pressure profile. It has been studied theoretically and experimentally the effects of surface waviness over the load carrying capacity of finite slider bearing. The author recorded enhanced load carrying capacity in the presence of surface waviness on the stationary pad.

**Figure 1:**
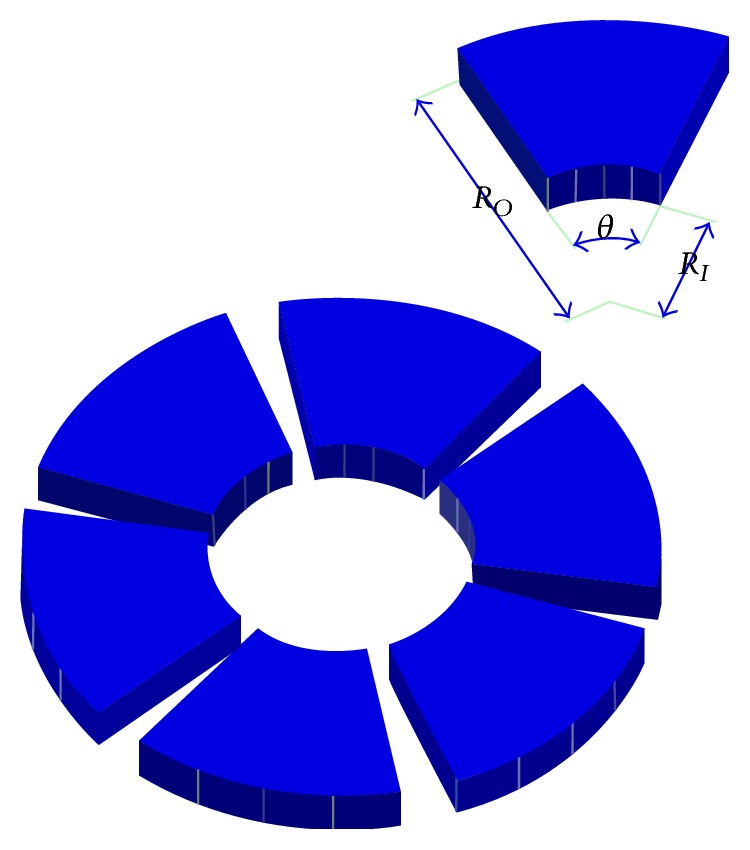
Thrust bearing pads and their characteristic dimensions.

The shape of the converging wedge influences the bearing performance significantly [13–15]. Investigations made by [11, 16] infinitely wide rough slider bearings isothermally for exponential, hyperbolic, and secant film shapes using couple stress fluids. The authors have reported that the increase in pressure is more for the exponential and hyperbolic sliders [16]. Moreover, investigators [17, 18] have studied the THD behavior of a slider bearing having a pocket and reported that the maximum pressure is higher for the pocketed bearing in comparison to plane slider bearing. [19, 20] studied the influence of film shape on the performance of longitudinally rough, infinitely wide slider bearing for isothermal conditions and reported better load carrying capacity with exponential, secant, and hyperbolic film shapes in comparison to the inclined plane film shape. Calculation model of the thrust bearing is built with the assumptions made in the previous chapters. It is prepared in the form of a sector shaped pad, which is totally immersed in the oil and supported on a supporting structure (different systems can be applied). Load is transferred from the rotating runner through the oil film to the bearing pad and the support. Rotational repeatability of the system is used so the model can be limited to a single sector. In the present work, a sector shaped six pad thrust bearing and its characteristic dimensions are shown in Figure 1. At normal speeds the surface of pad and runner are separated by a thin film lubricant. Bearing geometry and properties are shown in tabulated form in Table 1.

## 2. Reynolds Equation

The following assumptions are made in the analysis.

- Steady-state conditions exist in the oil film.
- The lubricant is incompressible.
- The lubricant is Newtonian in nature.
- Flow in the convergent wedge is laminar.
- Pressure and shear effects on the viscosity are negligible.

The analysis of hydrodynamic thrust bearings has been based on the Reynolds' equation for the pressure distribution. With the increase in capacity of computers, numerical models including the influences of viscosity variations along and across the lubricating film have been developed. The Reynolds equation is used to calculate the pressure field in the oil film. The variation of viscosity across the thickness of the oil film is neglected; Reynolds equation for a sector shaped thrust bearing pad, with an incompressible lubricant under steady state condition as reported by [3]. Equation (1) presents cylindrical coordinates of Reynolds equation. Consider

(1) $$\begin{array}{c} \frac{\partial }{\partial r}\left(\frac{{rh}^{3}}{\mu }\frac{\partial P}{\partial r}\right)+\frac{1}{r}\frac{\partial }{\partial \theta }\left(\frac{h^{3}}{\mu }\frac{\partial P}{\partial \theta }\right)=6\omega r\frac{\partial h}{\partial \theta }. \end{array}$$

This equation can be converted into nondimensional form by putting the following substitutions:

(2) $$\begin{array}{c} R^{*}=\frac{r}{R_{0}};\;\;\theta^{*}=\theta ;\;\;H^{*}=\frac{h}{H_{0}};\\ \mu^{*}=\frac{\mu }{\mu_{I}};\;\;P^{*}=\frac{{PH}_{0}^{2}}{{12\pi N\mu }_{I}R_{0}^{2}}. \end{array}$$

When the above substitutions are made after some simplifications the equation in its nondimensional form is as follows:

(3) $$\begin{array}{c} \frac{\partial }{{\partial R}^{*}}\left(\frac{H^{{*}^{3}}}{\mu^{*}}R^{*}\frac{{\partial P}^{*}}{{\partial R}^{*}}\right)+\frac{1}{R^{*}}\frac{\partial }{{\partial \theta }^{*}}\left(\frac{H^{{*}^{3}}}{\mu^{*}}\frac{{\partial P}^{*}}{{\partial \theta }^{*}}\right)=\frac{R^{*}{\partial H}^{*}}{{\partial \theta }^{*}}. \end{array}$$

## 3. Equation for Film Thickness

For sector shape geometry, film thickness is expressed in terms of (*r*-*θ*) coordinates. The compact film thickness expression reported by [15] considers variation in circumferential and radial direction. The oil film shape has been obtained using (4)

(4) $$\begin{array}{c} H=H_{0}+H_{s}\left(1-\frac{\theta }{\theta_{t}}\right). \end{array}$$

Converting above equation into nondimensional form by dividing above equation by *H*_0_ we get

(5) $$\begin{array}{c} H^{*}=1+\frac{H_{s}}{H_{0}}\left(1-\frac{\theta }{\theta_{t}}\right). \end{array}$$

## 4. Load Carrying Capacity (LCC)

Once the pressure distribution is determined, the load capacity can be calculated [12]. In nondimensional form, the load capacity is given by (6)

(6) $$\begin{array}{c} \text{LCC}=\frac{W}{Kr_{O}^{2}}=\overset{R_{o}}{\underset{R_{i}}{\int }}\overset{\theta_{t}}{\underset{0}{\int }}\left(PR\right)d\theta \;dR. \end{array}$$

## 5. Numerical Procedure

Numerical treatment of Reynolds equation (2D) using finite difference method for discretization of the sector shaped bearing pad is performed by considering different grid sizes in terms of (*M* × *N*) nodes and various convergence ratios “*K*” as given by (7)

(7) $$\begin{array}{c} K=\frac{H_{s+H_{0}}}{H_{0}}. \end{array}$$

**Figure 2:**
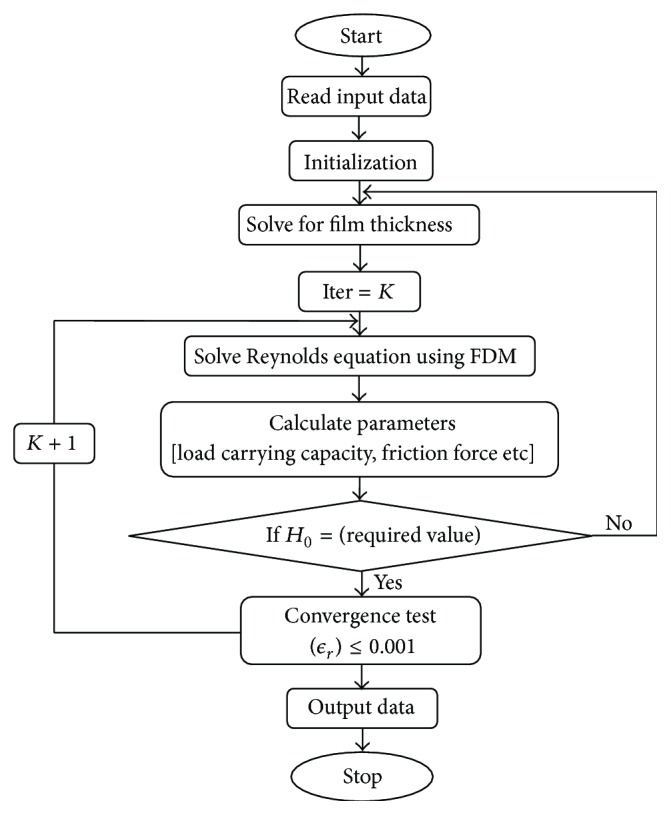
Flow chart for computation.

The finite difference equation is derived by approximating the derivatives in the differential equation through truncated Taylor series expansion for successive grid points. Writing the Reynolds equation in the finite difference form as in (8) results in set of linear algebraic equations, which are converted into the matrix form for the solution using Gauss-Seidel scheme for iteration along with the relevant boundary conditions and hence the nodal pressure (dimensionless) is computed. This will determine the nondimensional pressure at each node. The iteration will repeat until the oil pressure is converged as per the algorithm is shown in Figure 2, and the convergence criteria used for nodal pressure are given in (9). Consider

(8) Pi+1,j∗3Hij∗2μij∗Rij∗Hi+1,j∗−Hi−1,j∗4Δ  θ∗2Hij∗3μij∗2Rij∗μi+1,j∗−μi−1,j∗4Δθ∗2 +Pi−1,j∗Hij∗3μij∗2Rij∗μi+1,j∗−μi−1,j∗4Δθ∗2      −3Hij∗2μij∗Rij∗Hi+1,j∗−Hi−1,j∗4Δθ∗2 +Pi,j+1∗Hij∗32ΔR∗μij∗+Hij∗3Rij∗ΔR∗2μij∗      −Hij∗3Rij∗μij∗2μi,j+1∗−μi,j−1∗4ΔR∗2 +Pi,j−1∗Hij∗3Rij∗ΔR∗2μij∗−Hij∗3μij∗      +Hij∗3Rij∗2ΔR∗μij∗2μi,j+1∗−μi,j−1∗2ΔR∗ +Pi,j∗2Hij∗3Rij∗ΔR∗2μij∗+2Hij∗3Δθ∗2μij∗Rij∗=Ri,j∗Hi+1,j∗−Hi−1,j∗2Δθ∗.

The calculation treatment uses these pressure values along the numerical methods (as one-third Simpsons rule) for integration in order to calculate the load carrying capacity (LCC). A very tight tolerance value is considered here to ensure that the numerical derivative calculated by the algorithm shown is precise. Consider

(9) $$\begin{array}{c} \overset{M-1}{\underset{I=2}{\sum }}\overset{N-1}{\underset{I=2}{\sum }}\frac{\left|P_{I,J}^{\text{new}}-P_{I,J}^{\text{old}}\right|}{\left|P_{I,J}^{\text{new}}\right|}\leq \epsilon_{r}, \end{array}$$

where “*ϵ*_*r*_” is the tolerance limit. In this study a constant value of minimum film thickness 75 *μ*m has been kept under consideration [5]. The result of grid refinement study is found matching with the work reported [5]. Number of mesh sizes is there and their corresponding results in terms of pressure distribution and film thickness are presented in this paper.

## 6. Results and Discussion

**Figure 3:**
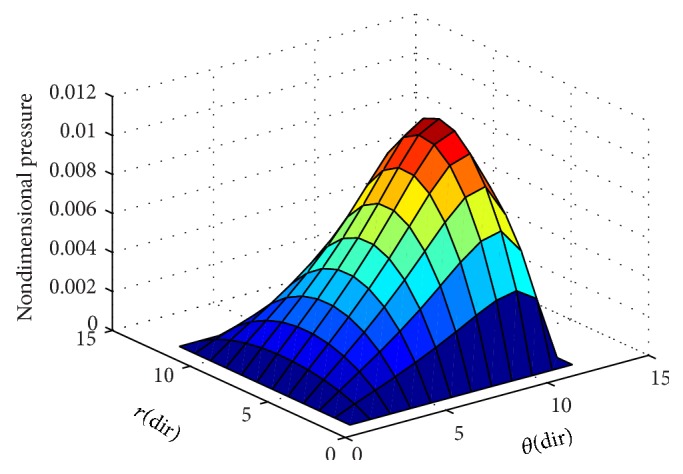
Nondimensional pressure distribution with grid size (12 × 12) in radial and circumferential directions.

**Figure 4:**
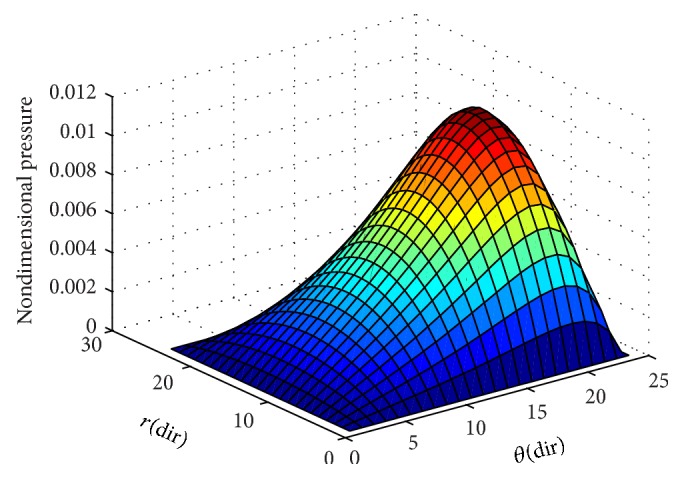
Nondimensional pressure distribution with grid size (24 × 24) in radial and circumferential directions.

**Figure 5:**
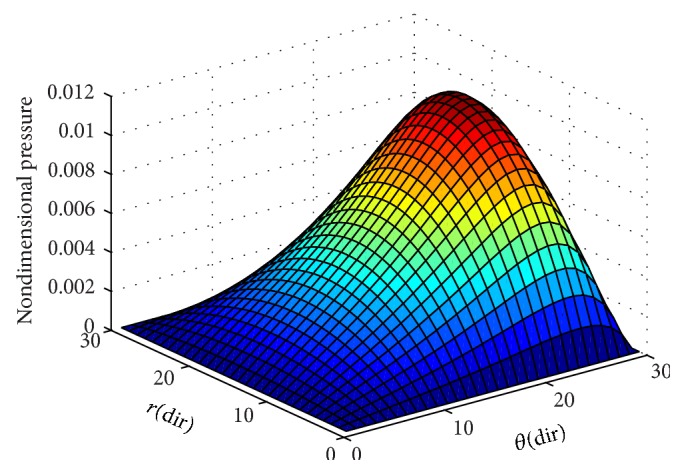
Nondimensional pressure distribution with grid size (30 × 30) in radial and circumferential directions.

**Figure 6:**
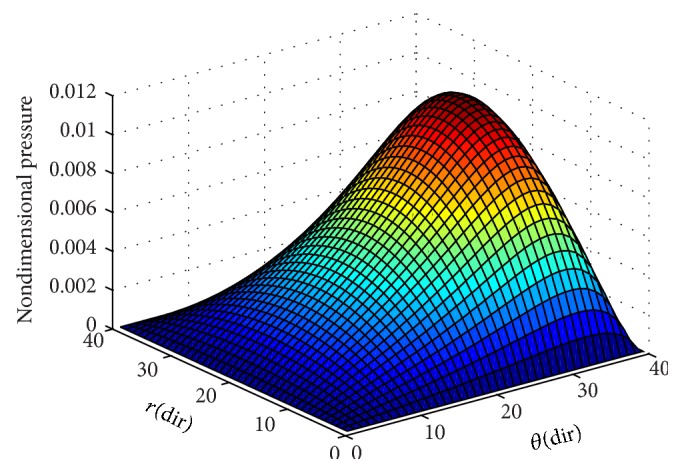
Nondimensional pressure distribution with grid size (40 × 40) in radial and circumferential directions.

**Figure 7:**
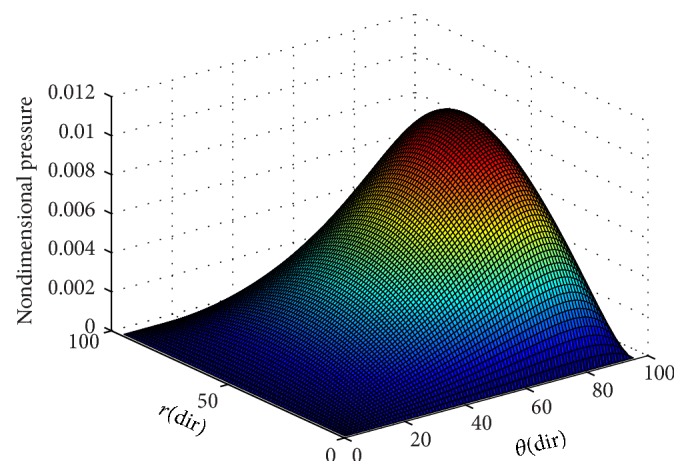
Nondimensional pressure distribution with grid size (96 × 96) in radial and circumferential directions.

**Figure 8:**
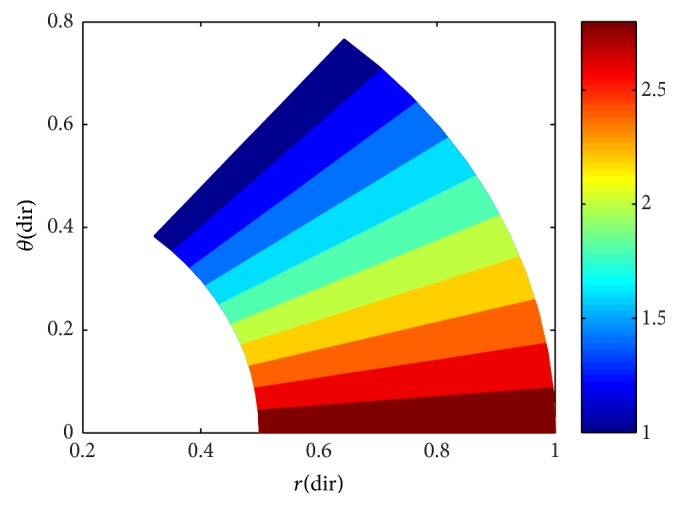
Nondimensional film thickness.

**Figure 9:**
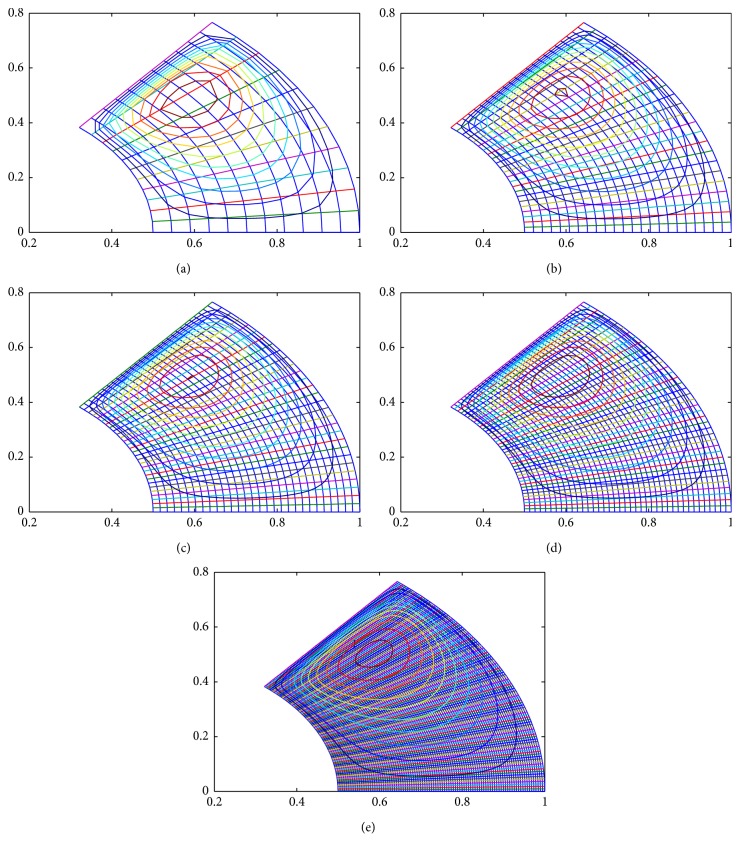
(a) Nondimensional pressure distribution contour on 12 × 12 grid size; (b) nondimensional pressure distribution contour on 24 × 24 grid size; (c) nondimensional pressure distribution contour on 30 × 30 grid size; (d) nondimensional pressure distribution contour on 40 × 40 grid size; (e) nondimensional pressure distribution contour on 96 × 96 grid size.

To ensure numerical accuracy, the pressure distribution as shown in Figure 9 satisfies the 0.1% convergence limit. In Figure 9, it is clear that the magnitude of pressure generation in the oil film for a sector shaped pad is changing from small grid size to larger one. In the leading edge side, the pressure on the pad surface is small; meanwhile, large pressure generation occurs on the pad surface in the vicinity of the trailing edge. The maximum values of pressure are located nearer to the trailing edge because of the peak pressure is slightly towards the trailing edge. The save minimum film thickness has been a limiting parameter used by analysis. The nondimensional oil film thickness distribution along the circumferential direction for center line and outer arc are shown in the Figure 8; grid independent study plays an eminent role in order to find the better solution of the numerical model. As we go on increasing the grid size from 12 × 12 to 96 × 96, the significant change comes in practice. Various 3D meshes of nondimensional pressure distribution are shown from Figures 3, 4, 5, 6, and 7. The results generated by the researchers [17, 18] are closely matching the path with grid size of 4 × 4, 8 × 8 and so forth. The results computed by [12], with the limited grid size of 9 × 9, are also showing good agreement with the present higher order grid size. In general the results are showing monotonic increase in accuracy and stability while shifting from course meshes to fine meshes.

**Figure 10:**
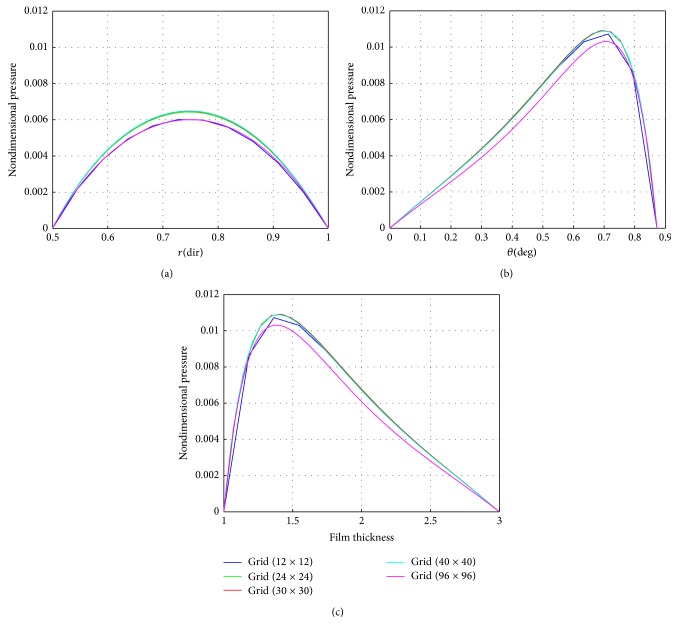
(a) Nondimensional pressure distribution for different grid sizes along the radial direction for sector shaped oil film; (b) nondimensional pressure distribution for different grid sizes along the circumferential direction for sector shaped oil film; (c) nondimensional Pressure distribution along and across the centre line of flow direction for different grid sizes of sector shaped oil film.

The maintained sustainable oil film thickness is to enhance the load bearing capacity. In the present case, the minimum oil film thickness is assumed to be a constant value of 75 *μ*m. A film profile is called a global optimum, if for a given set of operating conditions and minimum film thickness it can produce the top load carrying capacity among all possible film profiles. This is due to accommodation of the oil film thickness. Detailed results of pressure generation, film thickness values of Reynolds equation are reported in the work of [12, 15, 17, 18]. From the graphical interpretation shown in Figure 10(a), it is clearly understood that the nondimensional pressure distribution increases gradually from zero to a maximum value at centre of the pad almost in all cases of grid sizes along the radial direction. In Figure 10(b), it is noted here that the nondimensional pressure distribution is intensifying towards the trailing edge of the pad, but varies in magnitude in varying grid sizes. In Figure 10(c), it is observed that the nondimensional pressure distribution is much small and it abruptly reaches to the peak value at the minimum film thickness and thus counteracts the external load of the sliding surface. The results obtained are shown in Table 2 using a scaling factor “*S*” for computing the pressure at different grid levels. A relationship is developed to get the change in percentage in terms of a parameter “*ψ*” based on the reference grid at 9 × 9 grid size used by [12]. It is evident from the present study that there is a 12% increase in pressure values with the incorporation of grid independence.

The location of the peak pressure zone is also changing in terms of (*r*, *θ*) coordinates with respect to changing of grid sizes from courser to refine grids, hence on the basis of the present investigation, it is possible to find the exact location, where the pressure probes in the matrix form can be introduced in order to get the effective pressure values on the surface of the pad.

## 7. Validation 

It is clear that the present study is in close agreement with some of the works available in open literature [12, 15, 17, 18]. Since the present work is reporting the grid independence of pressure beyond 9 × 9 grid size, this feature is limitedly reported by [12, 17, 18]. The results for values of pressure distribution, therefore, limit the authors regarding one-to-one comparison for the refined mesh size with the previous works of [12, 15, 17, 18].

## 8. Conclusion 

This work analyzes the hydrodynamic performance characteristics of thrust bearing sector shaped pad taking full scale pressure generation effects. The governing equations are broken using FDM, expressed in their nondimensional form which finally has been solved for pressure distribution using appropriate boundary conditions. A numerical solution is proposed and an algorithm has been developed along with a numerical code. When the mesh size changes it has been observed that improvements in accuracy of the results were significant. The pressure value has been changed considerably with the embodiment of grid refinement analysis. It is evident from the present work that maximum average value of pressure is 12% greater than the results obtained by using coarse grid. At 24 × 24 grid the analysis shows an independent behavior of results, and it does not show further significant improvements, although result changes (albeit insignificantly) when the grid is further refined beyond 24 × 24. An important design parameter has been coined in the form of “*ψ*” during the present work. This will provide experimentalists a logical procedure for location of the pressure sensors.

## Figures and Tables

**Table 1:** Thrust bearing geometry and properties.

	Grade	Quantity
Description		
Inner radius		57.15 mm
Outer radius		114.3 mm
Number of pads		6
Pad angle		50.0°
Pivot angle		30.0°
Pad thickness		28.58 mm
Operating conditions		
Axial load		52265 N
Shaft speed		1500 rpm
Inlet temperature		40°C
Oil Properties		
Oil type	VG46	
Viscosity at 40°C		39.0 mPas
Viscosity at 100°C		5.4 mPas
Density		855.0 kg/m^3^
Thermal conductivity		0.13 W/m/K

**Table 2:** Effect of grid size on the results with grid refinement.

Grid size (*M* × *N*)	Pressure (ND) *P* ^*^	Pressure = *S* × *P* ^*^ Where “*S*” = 1000	*ψ* = |(*P*_newgrid_ − *P*_Refrence-grid_)/*P*_newgrid_|
12 × 12	0.0110	11.0	0.0542
24 × 24	0.0119	11.9	0.1273
30 × 30	0.01261	12.61	0.1751
40 × 40	0.01260	12.60	0.1765
96 × 96	0.01255	12.55	0.1713

Where “*S*” is a scaling factor which makes it convenient for noticing pressure values at different grid sizes.

## Nomenclature

*ε*_*r*_: Convergence criteria for computation
*H*_0_: Minimum oil film thickness (*μ*m)
*H*_*s*_: Amount of taper
*M*: Number of grid points along radial direction
*N*: Number of grid points along circumferential direction
*dθ*: Angular division of the grid (radian)
*dr*: Radial division of the grid (m)
*θ*_*t*_: Angular extent of the pad in degrees
*i*: Index of node in radial direction
*j*: Index of node in circumferential direction
*P*: Hydrodynamic pressure (N/m^2^)
*R*_*O*_: Outer radius of the pad (m)
*R*_*I*_: Inner radius of the pad (m)
*ρ*: Density of lubricating oil (kg/m^3^)
*μ*: Viscosity (Pas)
*ω*: Angular velocity of shaft (rad/s)
*w*: Load on bearing (k N)
*P*^*^: Non dimensional Pressure
*R*^*^: Non dimensional Radial coordinate
*θ*^*^: Non dimensional circumferential coordinate
*μ*^*^: Non dimensional viscosity
*H*^*^: Non dimensional oil film thickness
*ψ*: Grid refinement calculation Parameter
*ε*: Percentage tolerance limit for grid size variations
*K*: Convergence ratio.
